# Regional Differences in Islet Distribution in the Human Pancreas - Preferential Beta-Cell Loss in the Head Region in Patients with Type 2 Diabetes

**DOI:** 10.1371/journal.pone.0067454

**Published:** 2013-06-24

**Authors:** Xiaojun Wang, Ryosuke Misawa, Mark C. Zielinski, Peter Cowen, Junghyo Jo, Vipul Periwal, Camillo Ricordi, Aisha Khan, Joel Szust, Junhui Shen, J. Michael Millis, Piotr Witkowski, Manami Hara

**Affiliations:** 1 Department of Surgery, The University of Chicago, Chicago, Illinois, United States of America; 2 Institute of Hepatobiliary Surgery, Southwest Hospital, Third Military Medical University, Chongqing, China; 3 Department of Medicine, The University of Chicago, Chicago, Illinois, United States of America; 4 Laboratory of Biological Modeling, National Institute of Diabetes and Digestive and Kidney Diseases, National Institutes of Health, Bethesda, Maryland, United States of America; 5 Cell Transplant Center, University of Miami, Miami, Florida, United States of America; St. Vincent's Institute, Australia

## Abstract

While regional heterogeneity in islet distribution has been well studied in rodents, less is known about human pancreatic histology. To fill gaps in our understanding, regional differences in the adult human pancreas were quantitatively analyzed including the pathogenesis of type 2 diabetes (T2D). Cadaveric pancreas specimens were collected from the head, body and tail regions of each donor, including subjects with no history of diabetes or pancreatic diseases (n = 23) as well as patients with T2D (n = 12). The study further included individuals from whom islets were isolated (n = 7) to study islet yield and function in a clinical setting of islet transplantation. The whole pancreatic sections were examined using an innovative large-scale image capture and unbiased detailed quantitative analyses of the characteristics of islets from each individual (architecture, size, shape and distribution). Islet distribution/density is similar between the head and body regions, but is >2-fold higher in the tail region. In contrast to rodents, islet cellular composition and architecture were similar throughout the pancreas and there was no difference in glucose-stimulated insulin secretion in islets isolated from different regions of the pancreas. Further studies revealed preferential loss of large islets in the head region in patients with T2D. The present study has demonstrated distinct characteristics of the human pancreas, which should provide a baseline for the future studies integrating existing research in the field and helping to advance bi-directional research between humans and preclinical models.

## Introduction

Insulin-secreting pancreatic beta-cells play a key role in glucose homeostasis and the pathophysiology of diabetes. Beta-cells are organized into distinct structures termed the islet of Langerhans together with other endocrine cells, which is a micro-organ and a functional unit. Recent studies on the histology of the human pancreas have shown distinct islet architecture with an increased fraction of alpha-cells intermingled with beta-cells, in contrast to rodent islets with the central core of beta-cells and less alpha-cells residing in the periphery [1]–[3].

In a series of recent works, we have demonstrated that islet architecture is size dependent in humans where such drastic morphological changes occur selectively in large islets (>∼100 µm in diameter), but small islets show similar architecture with mice [4]–[6]. Moreover, such changes in large islets are not an intrinsic characteristic of human islets, but are also observed in mice under insulin resistance such as pregnancy, obesity, diabetes and inflammation [4], [7]. It is noted that the range of islet size distribution closely overlaps across various species with the maximum diameter being around 500–700 µm [4], [5], [7] suggesting that there are certain regulatory mechanisms that maintain optimal islet sizes in order to ensure their functional properties. Collectively, we propose that the histology of the pancreas should be studied in terms of the total islet size distribution rather than a selected regional analysis.

During embryogenesis, the pancreas arises from dorsal and ventral pancreatic protrusions from the primitive gut endoderm. Regions of the adult pancreas are anatomically referred to head, body and tail regions. The head region is located on the right side of the abdomen that attached to the duodenum. The body and tail region extends to the left side of the abdomen next to the spleen. Regional heterogeneities in the histology of islets have been well studied in rodents with largely similar observations that the density of beta-cell mass in the body and tail regions is higher than in the head region [8]–[15]. However, recently an elegant three-dimensional imaging study of mouse pancreas by Hörnblad et al reported a contradicting result of the regional differences in beta-cell volume in the order of duodenal > gastric > splenic lobe [16]. Some studies on functional differences of islets between ventral (head) and dorsal (body-tail) origin of the adult rat pancreas demonstrated that in the glucagon-rich dorsal islets, insulin secretion [9]–[12] and proinsulin biosynthesis [10] to glucose stimulation were significantly greater compared to the ventral islets. This result was further confirmed by *in vitro* experiment with the presence of excess exogenous glucagon in the culture media that compensated the functional differences [10].

In the head region of the human pancreas, past studies reported that 55–90% of the islet cell volume in this location was represented by pancreatic polypeptide (PP)-cells [17]–[21]. We have recently shown that the PP-cell rich area is more narrowly restricted and is largely segregated in the uncinate process (∼100% PP-cell rich) [22], which development varies individually due to early fetal development [23]. The PP-cell rich and poor areas coexist with a clear boundary in the head region and PP-cell distribution in the latter is similar to the rest of the pancreas. It is noteworthy that beta- and alpha-cell mass is significantly decreased in the PP-cell rich area compared to the PP-cell poor area. In the present study, the PP-cell rich area in the head region was first identified by PP-staining and excluded from subsequent quantification.

Our aim was to examine regional differences in islet distribution, cellular composition and architecture as well as glucose-stimulated insulin secretion, by highlighting the similarities and differences between humans and rodents. Here we report that: (1) Islet distribution/density is similar between the head and body regions, but is >2-fold higher in the tail region; (2) Similar cellular composition and architecture is present throughout the pancreas; and (3) There is no regional difference in glucose-stimulated insulin secretion in isolated islets. Further studies include the pathophysiology of T2D.

## Materials and Methods

### Ethics Statement

The use of human tissues in the study was approved by the Institutional Review Board at the University of Chicago.

### Human pancreas specimens

Human pancreata were generously provided by the Gift of Hope-Organ Procurement Organization in Chicago. Written informed consent from a donor or the next of kin was obtained for use of a sample in research. Specimens were collected within 12 hours of cold ischemia.

### Immunohistochemistry

Paraffin-embedded sections (5 µm) were stained with the following primary antibodies (all 1∶500): polyclonal guinea pig anti-porcine insulin (DAKO, Carpinteria, CA), mouse monoclonal anti-human glucagon (Sigma-Aldrich, St. Louis, MO), polyclonal goat anti-somatostatin (Santa Cruz, Santa Cruz, CA), polyclonal rabbit anti-pancreatic polypeptide (DAKO) and DAPI (Invitrogen, Carlsbad, CA). The primary antibodies were detected using a combination of DyLight 488, 549, and 649-conjugated secondary antibodies (1∶200, Jackson ImmunoResearch Laboratory, West Grove, PA).

### Image capture and quantification

Microscopic images were taken with an Olympus IX8 DSU spinning disk confocal microscope (Melville, NY) with imaging software StereoInvestigator (SI, MicroBrightField, Williston, VT). A modified method of “virtual slice capture” was used [24]–[26]. Briefly, the SI controls a XYZ-motorized stage and acquires consecutive images, which creates a high-resolution montage composed of images obtained from multiple microscopic fields of view. The entire tissue section was captured as “a virtual slice” using a 10× objective. Each virtual slice taken at four fluorescent channels were further merged into one composite. Quantification of cellular composition (i.e. each area of beta-, alpha-, and delta-cell populations, and islet area by automated contouring of each islet) was carried out using a macro custom-written for Fiji/ImageJ (http://rsbweb.nih.gov/ij/). To measure coordinates of each islet-cell type, DAPI fluorescent signals were converted to 8-bit masks and watershed to obtain masks of individual nuclei. The pixels surrounding nuclei masks were quantified with respect to each endocrine hormone staining (i.e. insulin, glucagon, or somatostatin) to identify which hormone was most prevalent around each nucleus and to record islet-cell coordinates. DAPI signals outside of islets were not included. Based on the coordinates of every cell within individual islets, neighborhood of each cell was identified and contact probabilities between cell types were calculated [5]. MATLAB (MathWorks, Natick, MA) was used for mathematical analyses.

### Human islet isolation

Human pancreata from multi-organ donors with consent for research and/or clinical use were processed at the Human Islet Cell Processing Facility of the Diabetes Research Institute at the University of Miami. Islets were isolated using a modified automated method [27], [28] Pancreas was cut at the neck dividing the organ into two sections, i.e. the head and the body-tail region. Two 16-gauge catheters were inserted into the main pancreatic duct in both pancreas blocks and collagenase solution was perfused. Each block was cut into several pieces, transferred to digestion chambers separately and processed simultaneously. Digested pancreatic tissues were collected and purified with either only continuous or continuous following discontinuous density gradient using a COBE 2991 Cell Processor (Gambro Laboratories, Denver, CO) at 4°C. Islet yield and particle count were determined by dithizone staining and expressed as islet equivalent number (IEQ) and islet particulate number (IPN), respectively.

### Glucose-Stimulated insulin release

Function of isolated islets based on insulin release in response to glucose was determined using the simulation index (SI), i.e. the ratio between stimulated (28 mM glucose) and basal (2.8 mM glucose) insulin releases in culture media for 2 h at 37°C. Supernatant was collected and insulin concentrations were assessed by ELISA (Alpco, Salem, NH).

### Statistical analysis

Data are expressed as mean ±SEM. Pairwise t-tests were used to compare mean percent distributions of beta-, alpha- and delta-cell populations across the pancreas regions: head, body, and tail. Corrections to p-values were made for multiple testing using the Bonferroni method (R: A language and environment for statistical computing) [29]. Differences were considered to be significant at *P*<0.05.

## Results

### Large-scale image capture and computer-assisted quantification of islet size distribution and cellular composition in whole tissue sections

**Figure 1:**
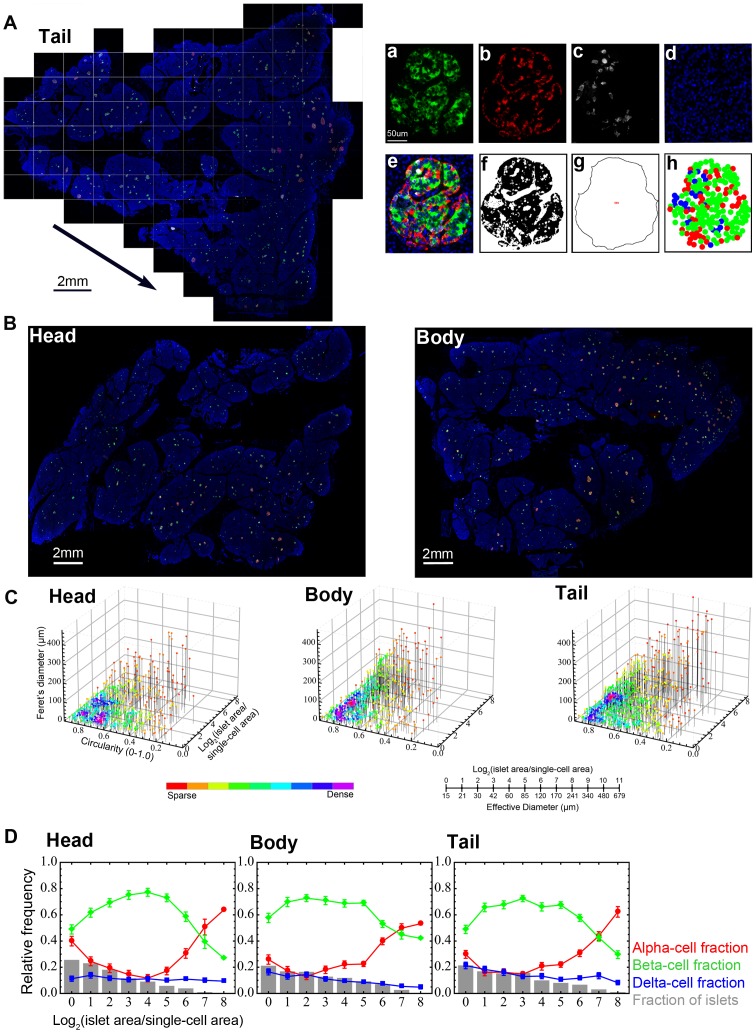
Large-scale image capture and computer-assisted semi-automated analysis of the whole tissue section. **A**: Virtual slice view of a human pancreatic section (tail region; ND21: 59-yr old male) immunostained for insulin (green), glucagon (red), somatostatin (white) and nuclei (blue). A series of contiguous images of a specimen is collected (illustrated as boxed panels) and merged into a single image montage (i.e. virtual slice; arrowed). A composite is made by merging four overlapping virtual slice images. Regional views of each channel are shown on the right. **a.** beta-cells, **b.** alpha-cells, **c.** delta-cells, and **d.** nuclei. **e.** A composite of all three endocrine cells and nuclei. Note that there is no overlap among the endocrine cell fractions. **f.** Total endocrine cell area shown as a converted 8-bit mask after automatic thresholding. **g.** Total islet area that includes unstained fractions such as intraislet capillary. **h.** Reconstructed endocrine cell distribution within each islet based on the captured center coordinates of each cell type within the given islet, which can be used to count the number of each endocrine cell type and analyze cellular composition and geographic islet architecture. **B**: Virtual slice views of the head and body region. **C**: Three-dimensional visualization of islet size (area) and shape (circularity and Feret's diameter) distribution in the head, body and tail region. Each dot represents a single islet/cluster. The density of islets is color-coded from sparse to dense. The conversion between logarithmic islet area and effective diameter (µm) is shown. **D**: Quantitative analysis of individual islet size distribution and cellular composition. Relative frequency of islet size (gray bar) and ratios of beta (green), alpha (red), and delta (blue) cells within islets are plotted against islet size; means ±SEM.

We first examined islet size distribution and cellular composition in the head, body and tail regions of the pancreas from cadaveric specimens with no history of diabetes or pancreatic diseases (n = 23; male: 43.9±5.3 yr, BMI 23.9±1.5; female: 49.2±1.9 yr, BMI 28.1±2.1). Clinical information about the pancreas tissue-donors is summarized in Table 1. Representative histology of a tail region from a 59-yr old male (ND21) is shown in Fig. 1A. We have applied a large-scale analysis of the major endocrine cell populations (beta-, alpha- and delta-cells) in the whole tissue section using a semi-automated computer assisted method [24]–[26], which provides the entire distribution of endocrine cells (from single cells to islets composed of thousands of cells). A composite is made by merging four overlapping virtual slice images as shown in Fig. 1A.a–e. Total endocrine cell area (Fig. 1A.f) and islet area that includes unstained fractions such as intraislet capillary are measured using converted 8-bit masks after automatic thresholding (Fig. 1A.g). Endocrine cell distribution within each islet is reconstructed based on the captured center coordinates of each cell type within the given islet (Fig. 1A.h), and this parameter is used to count the number of each endocrine cell type and analyze cellular composition and geographic islet architecture. Figure 1B shows virtual slice views of the head and body region from the same subject. In addition to each islet area, we also measure circularity (which reports the roundness of a structure where 1.0 represents a perfect circle) and Feret's diameter (the longest distance within a structure) so that islet size and shape distribution are visualized three-dimensionally as shown in Fig. 1C. Each dot represents a single islet/cluster. The density of islets is color-coded from sparse to dense. Note that islet size is presented as a logarithmic scale considering the high number of small islets and the low number of large islets. The conversion between logarithmic islet area and effective diameter (µm) is provided. Greater density of islets in the tail region is clearly demonstrated. Quantitative analysis of individual islet size distribution and cellular composition is shown in Fig. 1D. Relative frequency of islet size (gray bar) and ratios of beta (green), alpha (red), and delta (blue) cells within islets are plotted against islet size; means ±SEM. Note that in human islets, particularly in large islets, the fraction of alpha-cells increases. These large islets typically exhibit relatively intermingled architecture of beta and non-beta cells [3], [4]. Cellular composition of islets is similar across all the pancreatic regions.

**Table 1:** Subject information for morphological analysis.

Subject	Gender	Age	BMI	COD*/du**
**ND1**	M	15	16.0	Anoxia
**ND2**	M	20	28.7	Head trauma
**ND3**	M	20	21.2	Head trauma
**ND4**	F	37	21.7	Cerebrovascular/stroke
**ND5**	F	41	34.0	Stroke
**ND6**	F	45	33.4	Pulmonary thromboembolism
**ND7**	M	46	18.5	Anoxia
**ND8**	F	46	37.9	Anoxia
**ND9**	F	47	25.0	Anoxia
**ND10**	F	49	20.2	Anoxia
**ND11**	M	50	32.0	Head trauma
**ND12**	M	50	23.0	Cerebrovascular/stroke
**ND13**	M	51	26.0	Anoxia
**ND14**	M	51	27.2	Cerebrovascular/stroke
**ND15**	F	51	21.0	Cerebrovascular/stroke
**ND16**	F	52	25.7	Cerebrovascular/stroke
**ND17**	F	53	24.2	Cerebrovascular/stroke
**ND18**	M	53	21.4	Head trauma
**ND19**	F	53	42.5	Pneumonia
**ND20**	F	54	25.1	Cerebrovascular/stroke
**ND21**	M	59	28.1	Cerebrovascular/stroke
**ND22**	F	62	26.7	Anoxia
**ND23**	M	68	21.3	Cerebrovascular/stroke
**D1**	F	26	40.4	Head trauma, du: 6 mo
**D2**	M	38	29.0	Stroke
**D3**	F	42	19.0	Cerebrovascular/stroke
**D4**	F	42	37.9	Anoxia, du: 12 yr
**D5**	M	60	30.4	Cerebrovascular/stroke, du: 23 yr
**D6**	M	61	22.4	Anoxia, du: 25 yr
**D7**	F	66	31.0	Anoxia, du: 3 yr
**D8**	F	67	29.0	Intracranial hemorrhage, du: 15 yr
**D9**	F	68	34.0	Cerebrovascular/stroke, du: 20 yr
**D10**	M	71	31.0	Head trauma
**D11**	F	72	29.0	Stroke, du: 15 yr
**D12**	M	81	23.0	Anoxia

### Regional differences in islet distribution and cellular compositions in human pancreas

**Figure 2:**
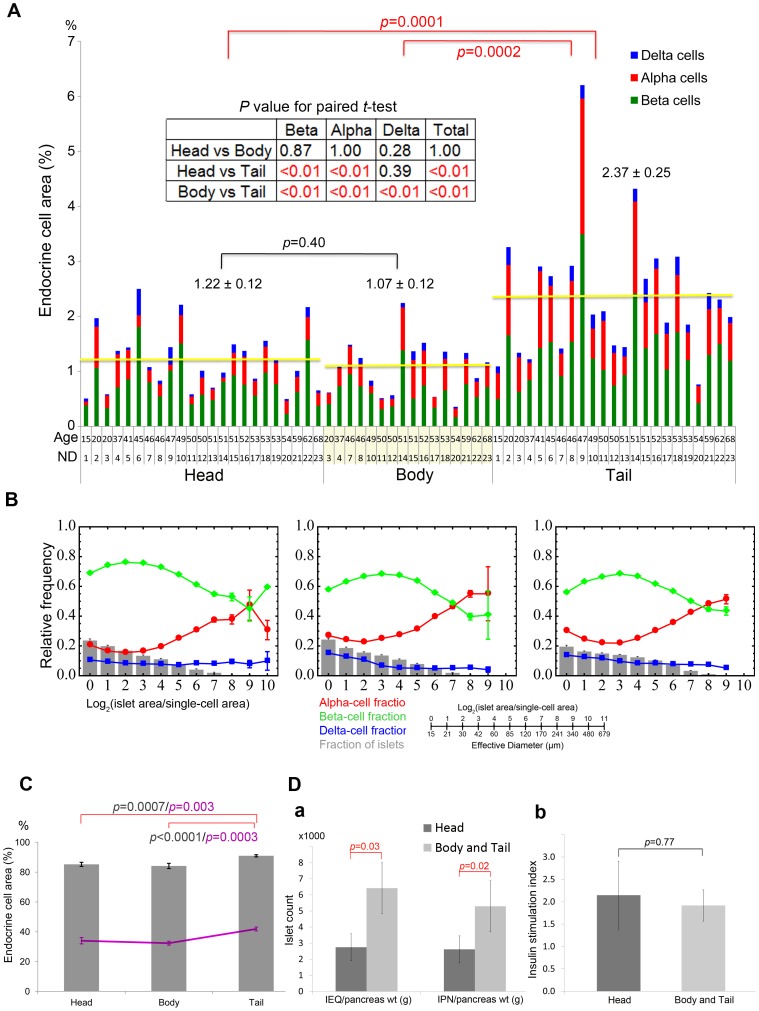
Regional differences in islet distribution and cellular compositions in human pancreas. **A**: Inter-specimen comparison of endocrine cell mass in the head, body and tail region. Total islet cell composition (beta-cells in green, alpha-cells in red, and delta-cells in blue) in individuals with no history of diabetes. Student's *t*-test compared the results between each region (with red and black bars). The regional differences were further confirmed in each individual by paired *t*-test (inset). **B**: Relative frequency of islet size (gray bar) and ratios of alpha (red), beta (green), and delta (blue) cells within islets are plotted against islet size; means ±SEM. **C**: The percent of large islets (>50 µm in diameter) in the total number of islets/clusters (line) and in the total area (bar). **D**: **a.** Regional differences of the yield of isolated islets (>∼50 µm in diameter; n = 7). IEQ: islet equivalent number and IPN: islet particulate number. **b.** Insulin secretory response measured as stimulation index.

Inter-specimen comparison of endocrine cell mass in the head, body and tail region is shown in Fig. 2A (n = 23). Regional differences in total islet cell composition (beta-cells in green, alpha-cells in red and delta-cells in blue) in each specimen were examined. There was no difference in total area of each endocrine cell mass normalized to the pancreas area between the head and body regions (1.22±0.12% and 1.07±0.12%, respectively), however, >2-fold higher islet density was observed in the tail region (2.37±0.25%) with a proportionate increase in each endocrine cell population. The regional differences were further confirmed in each individual by paired *t*-test (Fig. 2A inset). Quantitative analysis of mean values of individual islet size distribution and cellular composition in all specimens is shown in Fig. 2B. Islet size distribution and size-dependent cellular composition are similar throughout the pancreas. The percent of large islets (>50 µm in diameter) in the total number of islets/clusters and in the total area were further examined (Fig. 2C). Note that approximately 40% of the total number of these large islets consists of >80% of the total endocrine area. The tail region contains the significantly higher number of large islets (41.9±1.3%; Head: 34.0±2.2% and Body: 32.4±1.3%), which contributes to the higher percent of the total islet area compared to the head and body region (Tail: 91.0±0.8%; Head: 85.2±1.4%; Body: 84.1±1.8%). The higher density of islet distribution in the tail region was directly reflected to the islet yield in the clinical setting of islet isolation (Fig. 2D). Clinical information about the islet donors is summarized in Table 2. When islets were isolated concomitantly from two divided blocks from each donor pancreas (i.e. the head and the body-tail region cut in the neck of the pancreas, n = 7), the islet yield from the body-tail region (normalized to the regional pancreas weight as a block) was >2-fold higher than that of from the head region (Fig. 2D.a. IEQ/g: Head 2,747±838 and Body-Tail 6,422±1,582; IPN/g: Head 2,617±812 and Body-Tail 5,294±1,247). However, there was no regional difference of isolated islets in insulin secretory response to glucose *in vitro* (Fig. 2D. b. SI: Head 2.15±0.76 and Body-Tail 1.92±0.35).

**Table 2:** Subject information for islets isolation.

Subject	Gender	Age	BMI	Cause of Death
**1**	M	21	39.6	Head trauma
**2**	F	33	32.6	Head trauma
**3**	M	34	30.2	Intracranial hemorrhage
**4**	M	38	32.1	Intracranial hemorrhage
**5**	F	45	30.0	Head trauma
**6**	F	45	32.3	Intracranial hemorrhage
**7**	F	59	26.2	Intracranial hemorrhage

### Regional changes of islet distribution and cellular compositions in patients with T2D

**Figure 3:**
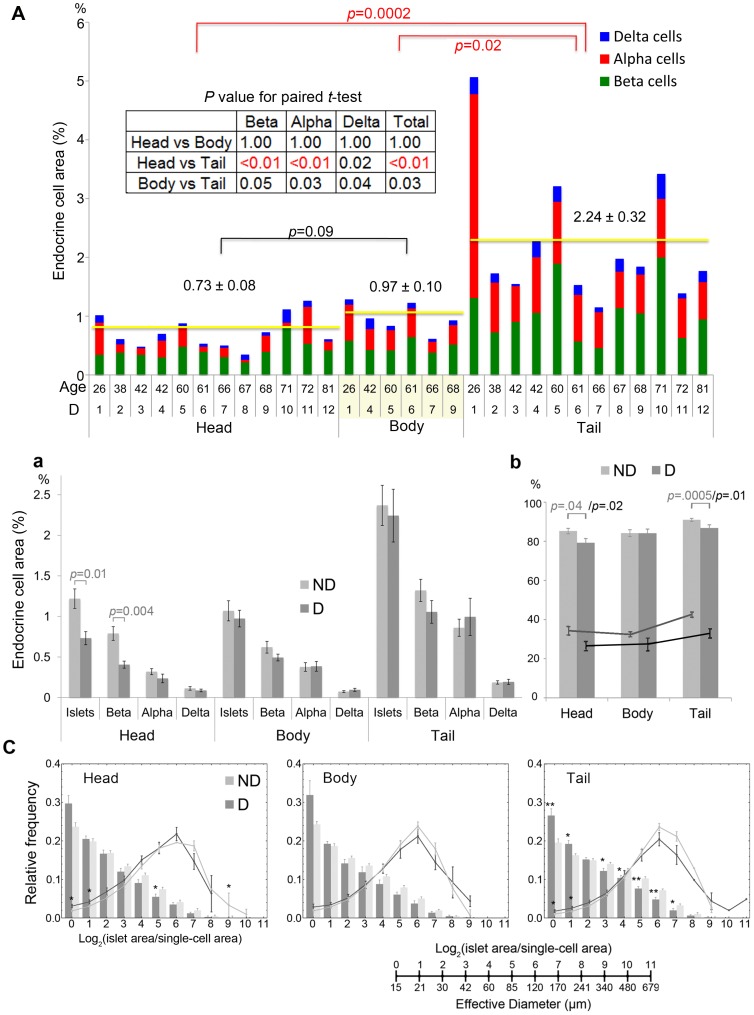
Regional changes of islet distribution and cellular compositions in patients with T2D. **A**: Inter-specimen comparison of endocrine cell mass in the head, body and tail region. Total islet cell composition (beta-cells in green, alpha-cells in red, and delta-cells in blue) in patients with T2D is shown. Student's *t*-test compared the results between each region (with red and black bars). **B**: **a.** Comparison between non-diabetic subjects (ND) and patients with T2D (D) in the total mass of islets and each endocrine cells (in gray and black bars, respectively). **b.** The percent of large islets (>50 µm in diameter) in the total number of islets/clusters (lines; ND in gray and D in black) and in the total area (bars; ND in gray and D in black). **C**: Changes in islet size distribution (bar) and contribution of each islet size bin to the total islet area (line) in patients with T2D (in black) compared to non-diabetic subjects (in gray).

Inter-specimen comparison of endocrine cell mass in the head, body and tail region in patients with T2D is shown in Fig. 3A (n = 12, Table 1; male: 62.2±7.2 yr, BMI 28.2±1.8; female: 54.7±6.7 yr, BMI 31.5±2.7). Similarly to non-diabetic subjects, while there was no difference in total endocrine mass between the head and body region (0.73±0.08% and 0.97±0.10%, respectively), it was >2-fold higher in the tail region (2.24±0.32%). Interestingly, further analysis on regional differences in each individual by paired *t*-test revealed significant beta-cell loss in the head region (Fig. 3A inset). Comparison between non-diabetic subjects (ND in gray) and patients with T2D (D in black) in the total mass of islets and each endocrine cells showed the preferential beta-cell loss in the head region in T2D patients, which led to the significant overall reduction in the total islet area (Fig. 3B.a). Comparison of the percent of large islets (>50 µm in diameter) in the total number of islets/clusters and in the total area revealed the preferential loss of large islets in the head and tail region in patients with T2D (Fig. 3B.b, Head: 26.5±2.4% and Tail: 32.2±2.4% in number; and Head: 79.2±2.2% and Tail 86.8±1.6% in area). Detailed analysis on islet size distribution (bar) and contribution of each islet size bin to the total islet area (line) in patients with T2D (D in black) compared to non-diabetic subjects (ND in gray) showed the overall leftward shift in patients with T2D in all three regions (Fig. 3C; although the body region was not affected).

**Figure 4:**
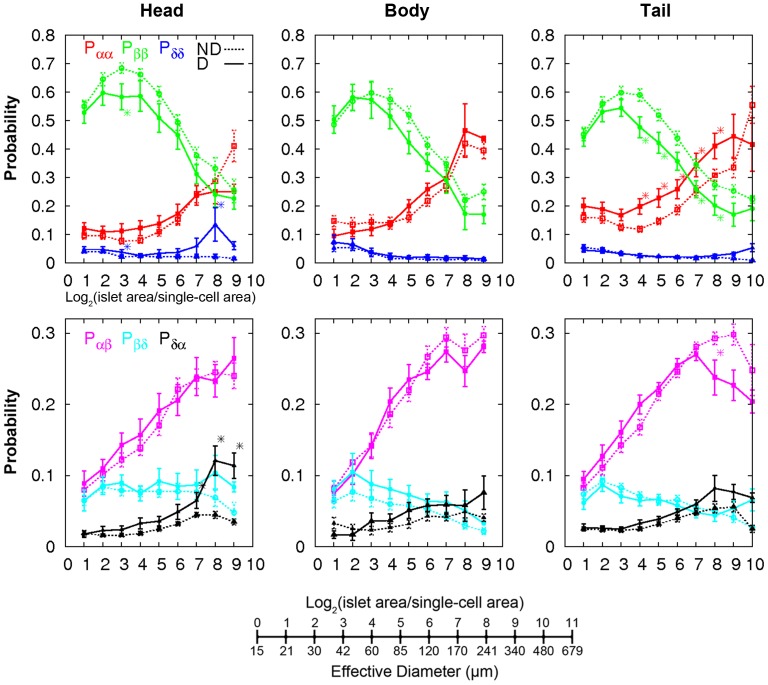
Changes of islet architectures in patients with T2D. Cell-cell contact probabilities calculated using fractions of specific contacts between neighboring cell types (e.g. P_αβ_ quantified the fraction of alpha- and beta-cell contacts among every neighboring cell contact). Non-diabetic (ND, dotted line) and Diabetic (D, solid line). Mean ±SEM. *P<0.05 from student's t-test between ND and D.

Lastly, changes of islet architectures were examined by calculating the probabilities of contact between cell types (Fig. 4) [5]. We first defined neighbors of each cell within an islet and counted the number of contacts of 6 combinations (i.e. alpha-alpha, beta-beta, delta-delta, alpha-beta, beta-delta, and alpha-delta cells). The contact probability depicts the relative frequency of specific cell-cell contacts. The results showed similar trends among pancreas regions. Islets in diabetic subjects had less contacts between beta-cells and more contacts between alpha-cells.

## Discussion

We examined regional heterogeneity in the human pancreas comparing head, body and tail regions from each individual to understand species differences, whereas past studies characterized the pancreatic regions to a limited extent using point-count morphometry [30]–[34]. In the present study, we have particularly applied large-scale image capture and computer-assisted quantification of islet size distribution and architecture that provides an unbiased representative view of an entire tissue section [5], [6], [26]. We report that largely similar to rodents, the tail region contains >2-fold higher islet distribution compared to the head and body region. Despite inter-subject variability, the regional differences were consistent in each individual as confirmed by paired *t*-test. The regional difference in islet density reflected the yield of isolated human islets (normalized to the regional pancreas weight) that it was >2-fold higher in the body-tail region compared to the head region. Note that here the islet size distribution is in a clinically relevant range (>∼50 µm in diameter). In contrast to rodents [8]–[16], cellular composition and architecture were similar in humans throughout the pancreas and no regional difference in glucose-stimulated insulin secretion was observed in isolated islets. Further analysis revealed the significant loss of beta-cells preferentially in the head region in patients with T2D. Overall reduction of beta-cell mass was observed in the body and tail region, however it was not statistically significant.

The present study may have an important implication for the future studies on tissue harvesting, particularly the need to identify anatomical regions of pancreas specimens. In cancer research, several studies report that the majority of pancreatic cancer involves the head region, whereas the less frequent tumors in the body and tail region are more malignant [35]–[39]. T2D is a major risk factor for the development of pancreatic cancer (following smoking and obesity) and the T2D epidemic has been suggested to be responsible, at least in part, for the increase in the incidence of pancreatic cancer [40], [41]. It is of great interest to identify factors and underlying mechanisms that lead the head region to be more susceptible for beta-cell loss in T2D as well as pancreatic cancer development.
